# “Fit to Kill”: A Case Report on Undiagnosed Temporal Lobe Epilepsy Leading to Homicide

**DOI:** 10.1192/bjo.2025.10829

**Published:** 2025-06-20

**Authors:** Indu Surendran, Scott Broadhurst, Inti Qurashi

**Affiliations:** 1 Mersey and West Lancashire NHS Foundation, Prescot, United Kingdom; 2 Merseycare Mental Health NHS Foundation Trust, Maghull, United Kingdom

## Abstract

**Aims:** Temporal Lobe Epilepsy (TLE) is the most common symptomatic cause of partial epilepsy worldwide. It can present with vague symptoms including affective disturbances, personality changes, sensory disturbances, altered consciousness etc., which can confound diagnosis. Overt focal or tonic-clonic seizures are present in only 60% patients. It manifests with psychotic symptoms in around 5%. This case report describes how undiagnosed TLE with psychotic symptoms led to grave consequences.

**Methods:** We present a 52-year-old male who was found to have killed his father and was subsequently diagnosed with TLE. This diagnosis was later found to be directly implicated in the act. When arrested on suspicion of homicide he presented with several psychotic symptoms and severe agitation in custody. He was then detained under the Mental Health Act to a high secure psychiatric hospital. He had no formal psychiatric or medical diagnosis prior to his arrest but it was noted that he had presented on multiple occasions to secondary neurology and psychiatric services over the preceding 10 years with vague but consistent symptoms which were labelled as night terrors. These included clouding of consciousness, affective disturbance, night terrors, suicidal ideation, and isolated aggressive outbursts. Following his hospital admission, he was assessed by neurologists and was diagnosed of TLE after his EEG clearly demonstrated abnormalities in line with the diagnosis. The gentleman’s psychiatric symptoms resolved entirely when he was treated adequately for TLE with an anti-epileptic (lamotrigine). At trial, he was found not guilty of murder by reason of insanity and received a hospital order with restrictions.

**Results:** TLE is a treatable neurological disorder but is associated with a risk of heightened violence especially if misdiagnosed. In this patient, repeated opportunities were missed in diagnosing and treating TLE in a timely manner. Potential differentials that can confound TLE diagnosis include night terrors, functional psychosis, anxiety disorders, affective disorders, substance misuse etc.

**Conclusion:** TLE can have far-reaching and life-altering consequences. To obviate these, it is recommended that repeated presentations with unexplained neurological and psychiatric symptoms in outpatient or emergency settings should trigger robust assessments and multi-specialty liaison. It would be beneficial to develop a protocol to trigger a thorough evaluation of suspected TLE cases with a lower threshold to investigate them than what is currently practised. This could involve earlier and wider utilisation of electro-encephalogram (EEG) (ambulatory or residential).

